# 下调HOTAIR通过提高PTEN表达逆转HCC827细胞吉非替尼耐药

**DOI:** 10.3779/j.issn.1009-3419.2020.103.13

**Published:** 2020-09-20

**Authors:** 阳 翟, 茜 陈, 玉珍 王, 旭 李, 丽娜 李

**Affiliations:** 1 710061 西安，陕西省肿瘤医院肿瘤内科 Department of Oncology, Tumor hospital of Shaanxi Province, Xi'an 710061, China; 2 710061 西安，西安交通大学第一附属医院生殖医学科 Department of Reproductive Medicine, The First Affiliated Hospital of Xian Jiaotong University, Xi'an 710061, China

**Keywords:** HOTAIR, PTEN, HCC827细胞, 吉非替尼, 耐药性, HOTAIR, PTEN, HCC827, Gefitinb, Drug resistance

## Abstract

**背景与目的:**

肺癌是发病率和死亡率最高的恶性肿瘤，其中80%以上为非小细胞肺癌。HOX转录反义RNA(HOX transcript antisense RNA, HOTAIR)异常表达于多种肿瘤组织，并参与调控肺癌的发生与发展，本研究旨在探讨下调HOTAIR对肺腺癌HCC827细胞对吉非替尼药物耐药的影响及其机制。

**方法:**

应用实时荧光定量PCR(quantitative real-time PCR, RT-qPCR)法检测HCC827细胞及HCC827吉非替尼耐药细胞(HCC827GR)中HOTAIR的表达情况，利用生物信息学分析预测HOTAIR靶基因，应用脂质体转染法将体外合成的针对HOTAIR的siRNA转染入HCC827GR细胞中，采用RT-qPCR及Western blot方法检测其HOTAIR及PTEN、PI3K、AKT的表达水平，同时采用MTT法检测各组细胞的吉非替尼半数抑制浓度(50% inhibitory concentration, IC_50_)，运用流式细胞术分析各组细胞凋亡率的变化。

**结果:**

RT-qPCR结果显示HOTAIR在HCC827GR耐药细胞及吉非替尼耐药患者血清中表达增高，利用生物信息学分析预测到PTEN的为HOTAIR潜在靶基因。RT-qPCR及Western blot结果显示HCC827GR细胞经转染HOTAIR siRNA后，HOTAIR表达降低(*P* < 0.05)，而PTEN表达升高，PI3K及AKT表达下降(*P* < 0.05)；与对照组比较，下调HOTAIR的HCC827GR细胞对吉非替尼的IC_50_值下降(*P* < 0.05)，细胞增殖能力下降、凋亡率升高(*P* < 0.05)。

**结论:**

下调HOTAIR表达可抑制HCC827GR细胞增殖，促进其凋亡，并可降低HCC827GR细胞的吉非替尼IC_50_，下调HOTAIR后PTEN表达升高，而PI3K及AKT表达下降提示下调HOTAIR可通过靶向调控PTEN/PI3K/AKT通路逆转HCC827GR细胞对吉非替尼的耐药。

## Abstract (en)

**Background and Objective:**

Lung cancer is the most common cancer worldwide with the highest morbidity and mortality, in which the non-small cell lung cancer accounts for 80% of all cases. The expression of (HOX transcript antisense RNA) HOTAIR were abnormal in a variety of tumor tissues and is involved in the regulation of the occurrence and development of lung cancer. The purpose of this study is to investigate the effect and mechanism of down-regulation of HOTAIR on gefitinib resistance of lung adenocarcinoma HCC827 cells by targeting PTEN.

**Methods:**

The HOTAIR downstream target gene was predicted by bioinformatics database. The small interfering RNAs (siRNA) which is corresponding to HOTAIR was transfected using Lipofectamine^TM^ 2000. Quantitative real-time PCR (RT-qPCR) and Western blot were used to detect the expression of HOTAIR, PTEN, PI3K and AKT in HCC827 and HCC827GR cells. MTT assay was used to detect the changes in drug resistance of HCC827GR cells. Flow cytometry analysis were used to test the cell proliferation and the rate of apoptosis.

**Results:**

The expression of HOTAIR increased in HCC827GR and the serum of NSCLC patients with gefitinib resistance (*P* < 0.05). Transfection of HOTAIR siRNA decreased the expression of HOTAIR (*P* < 0.05), and increased the expressions of PTEN (*P* < 0.05), while the expression of PI3K and AKT were decreased (*P* < 0.05). Compared with the blank control group, down-regulation of HOTAIR increased the sensitivity of HCC827GR cells to gefitinib. The cell proliferation ability was decreased and the apoptosis was promoted apparently (*P* < 0.05).

**Conclusion:**

Down-regulation of HOTAIR can suppress the cell growth and promote the apoptosis, and it can reverse the resistance of HCC827GR cells to gefitinib. Its potential mechanism may be related with the targeting of PTEN/PI3K/AKT pathway.

肺癌的发病率和死亡率均居全球及中国恶性肿瘤中的首位，其中约80%以上为非小细胞肺癌(non-small cell lung cancer, NSCLC)^[1]^，目前分子靶向治疗已成为NSCLC治疗的主要手段之一，其中以吉非替尼(Gefitinib)为代表的表皮生长因子受体酪氨酸激酶抑制剂(epidermal growth factot receptor tyrosine kinase inhibitors, EGFR-TKIs)具有突出的成效，但晚期NSCLC患者一线使用Gefitinib靶向治疗的中位疾病无进展生存时间(progression-free survival, PFS)仅为9.6个月^[2]^，因此，探索EGFR-TKIs耐药机制、寻找逆转耐药的途径成为了亟待解决的问题。

长链非编码RNA(long noncoding RNA, lncRNA)是长度超过200 nt的一类非编码内源性RNA，定位于细胞质及细胞核，因lncRNA缺乏明显的开放阅读框而不能编码蛋白质，但却能够通过转录、转录后及表观遗传学水平对肿瘤细胞的生物学特性进行调控^[3-5]^。HOX转录反义RNA(HOX transcript antisense RNA, HOTAIR)定位于人类染色体12q13.13，长度为2, 364bp，既往研究^[6-8]^表明，HOTAIR异常表达于多种肿瘤细胞，能够通过双向结合多梳蛋白抑制复合体2(polycomb repressive complex 2, PRC2)和赖氨酸特异性去甲基化酶1(lysine specific demethylase 1, LSD1)修饰靶基因组蛋白并使下游基因沉默，同时能够促进上皮-间质化过程并能同肿瘤抑制因子及微小RNA(microRNAs, miRNAs)相互作用，从而调控肿瘤细胞的增殖、分化及凋亡，并参与肿瘤的发生与发展。

既往研究^[9]^发现，HOTAIR在NSCLC患者组织中呈高表达，且参与了NSCLC的发生发展及侵袭转移的过程。Zhang等^[10]^研究发现HOTAIR能够通过激活PI3K/Akt信号通路与第10号染色体缺失的张力蛋白同源的磷酸酶基因(phosphatase and tensin homology deleted on chromosome ten, PTEN)结合，从而促进子宫内膜癌细胞增殖。Li等^[11]^发现在人喉部鳞癌中，HOTAIR可诱导PTEN的甲基化和下调，导致PI3K/AKT通路活性增加，从而促进肿瘤的增殖和转移。Chen等^[12]^在乳腺癌中也得出了一致的结果。提示我们HOTAIR能够通过激活PTEN/PI3K/AKT信号通路发挥调控肿瘤的作用。

本研究采用质体转染法将体外合成的针对HOTAIR的siRNA转染HCC827GR入细胞中，通过生物信息学预测HOTAIR的靶基因，并应用实时荧光定量PCR(quantitative real-time PCR, RT-qPCR)及Western blot方法检测HOTAIR及PTEN、PI3K、AKT的表达水平，同时采用MTT法检测各组细胞的吉非替尼半抑制浓度(50% inhibitory concentration, IC_50_)，运用流式细胞术分析各组细胞凋亡率及生长周期的变化。从体外实验证明下调HOTAIR表达可以通过调控PTEN/PI3K/AKT通路，抑制HCC827细胞增殖，促进其凋亡，并可以逆转HCC827GR细胞对吉非替尼的耐药，下调HOTAIR表达可能成为逆转NSCLC患者EGFR-TKIs耐药的新策略。

## 1 材料与方法

### 1.1 实验材料

收集2019年1月-2020年1月陕西省肿瘤医院确诊的NSCLC患者外周血血清7例，其中吉非替尼治疗有效患者4例，耐药患者3例，男性3例，女性4例，平均年龄(65.7±10.3)岁。人肺癌HCC827细胞株及HCCC827吉非替尼耐药细胞株(HCC827GR)由西安交通大学生物医学中心实验室保存，HCC827GR细胞为前期参考Fumiaki等^[13]^在文献中提供的方法通过低浓度吉非替尼持续诱导HCC827细胞构建。用CCK-8法检测该细胞对吉非替尼的敏感性，计算IC_50_在20 μmol/L左右，并且在不加药培养基培养的条件下，相关耐药表型稳定至少6个月，即为构建成功。

### 1.2 细胞培养

使用RPMI-1640培养基加20%胎牛血清(FBS)和100 U/mL的双抗(均购自美国Hyclone公司)，孵箱条件为37 ℃，5%CO_2_，饱和湿度的培养箱中(购自美FOR-MA公司)，0.25%胰酶+0.02% EDTA(购自美国Sigma公司)消化、传代。实验选用对数生长期的细胞。

### 1.3 生物信息

学分析利用Targetscan数据库(http://www.targetscan.org)及Starbase数据库(http://starbase.sysu.edu.cn)预测HOTAIR及PTEN的潜在相关结合位点。

### 1.4 siRNA转染

按实验需要分为6组：HCC827组、HCC827GR组、HCC827GR/HOTAIR-NC组、HCC827GR/si-HOTAIR组、HCC827GR/HOTAIR-NC+gefitinib组及HCC827GR/si-HOTAIR+gefitinib组。si-HOTAIR序列为5’-UUUUCUACCAGGUCGGUAC-3’，阴性对照(negative control, NC)序列为5’-UUCUCCGAACGUGUCACGUTT-3’(由上海吉玛制药技术有限公司合成)，转染前1天将(4-5)×10^4^细胞接种在24孔板上，培养于37 ℃、5%CO_2_、饱和湿度的培养箱中。第二天将稀释好的siRNA和lipofectamin^TM^ 2000(购自美国Invitrogen公司)试剂混匀形成siRNA/lipofectamin复合物，100 μL siRNA/Lipofectamin复合物加到含有细胞和培养基的培养板的孔中，放入CO_2_培养箱中37 ℃温育24 h-48 h。

### 1.5 RT-qPCR

检测HOTAIR、PTEN表达HOTAIR引物上游：5’-TAGGCAAATGTCAGAGGGTT-3’，下游：5’-ACACAAGTAGCAGGGAAAGG-3’; PTEN引物上游：5’-CTATTCCCAGTCAGAGGCGCTAT-3’; 下游：5’-TGAACTTGTCTTCCCGTCGTGT-3’; GAPDH:上游：5’-ATTGATGGAT GCTAFGAGTATT-3’，下游：5’-AGTCTTCTGGGTGGCA GTGA T-3’(由北京鼎国昌盛生物技术有限责任公司合成)。结果判读：扩增完毕后行溶解曲线分析，以GAPDH为内参，采用公式：采用公式：2^-∆∆Cq^[∆∆Cq = Cq(target gene)-Cq(GAPDH)]计算各指标的mRNA相对表达量。以上步骤重复3次。

### 1.6 Western blot

检测各组细胞PTEN、PI3K、AKT蛋白表达选择生长良好的细胞，加入蛋白裂解液提取蛋白并采用BCA法定量(BCA蛋白定量试剂盒购自陕西先锋生物科技有限公司)，取30 μg蛋白，加入等体积2×loading buffer，煮沸5 min。配制10% SDS-PAGE胶，将凝胶玻璃固定于电泳装置上，加入电泳缓冲液，将样品短暂离心，用微量上样器吸取适量样品，缓慢加入蛋白Marker及样品，之后在外槽中加入适量电泳缓冲液; 电泳、转膜、封闭后加入封闭液稀释好的一抗c-MET及β-actin(稀释比为1:2, 000)，密封条件下于4 ℃冰箱过夜。洗膜后加入封闭液稀释好的二抗(稀释比为1:2, 000)，室温下于摇床上温和摇动2 h。洗膜、发光，ECL显影5 min。采用图像采集系统对X线片结果进行扫描取图，利用Image J软件对所摄图片进行灰度值分析及比较。各组样品目的蛋白与相应内参的灰度值比值为其最终统计分析数据。每组重复3次。

### 1.7 MTT法检测细胞的药物敏感性

将HCC827GR细胞用0.25%胰酶+0.02% EDTA消化，接种于96孔板，置于37 ℃、5%CO_2_、饱和湿度的培养箱中培养24 h，每孔中加入不同浓度梯度的吉非替尼，各浓度设5孔，并设调零孔(只有培养液)和对照孔(培养基+药物溶解剂+细胞)。分别培养24 h、48 h、72 h后：每孔加入灭菌的5mg/mL MTT(美国Sigma公司)20 μL，继续培养4 h，弃去培养液，加入DMSO液(美国Sigma公司)150 μL/孔，水平振荡10 min使紫蓝色结晶物充分溶解后置于高通量多功能微板测试(492 nm)测定各孔光吸收值(以*A*表示)，以空白孔调零，取4孔平均值，计算出细胞抑制率IR=(1-*A*/A0)×100%[*A*：各药物浓度组光吸收值; *A*0：未加药物组(对照组)光吸收值]。

### 1.8 细胞凋亡率检测

采用Annexin V-PE/7-AAD双染法，应用流式细胞仪检测(购自美国BD公司)HCC827GR细胞凋亡率的变化。取对数生长期细胞，用0.25%胰酶+0.02% EDTA消化，接种于6孔板，培养至对数生长期。再使用0.25%胰酶(不含EDTA)消化细胞，进行细胞计数，使每个样品约(0.5-1)×10^6^个/mL细胞，加入结合缓冲液500 μL，重悬细胞; 室温、避光条件下加入7-AAD染液5 μL(陕西先锋生物科技有限公司)，室温避光孵育5 min-15 min，再加入Binding buffer 450 μL，混匀，使抗体与细胞充分结合，加入1 μL Annexin V-PE染液，孵育15min，1h内上机检测。

### 1.9 细胞周期检测

取对数生长期细胞，用0.25%胰酶+0.02% EDTA消化，接种于6孔板，培养至对数生长期。再次使用常规胰酶消化法制成单细胞悬液; 用含3%小牛血清的70%乙醇固定，4 ℃保存过夜; 取冷存的细胞，1, 000 r/min×8 min离心，去上清; PBS洗涤2次; 加入150 μL RNase(5 g/L)(美国Sigma公司)和150 μL PI溶液(50 μg/mL)(美国Sigma公司)于室温避光染色30 min; 使用流式细胞仪检测，分析G_0_期/G_1_期、S期、G_2_期/M期细胞百分比。

### 1.10 *Kaplan-Meier* Plotter生存分析

从TCGA数据库(http://tcga-data.nci.nih.gov/tcga)中下载肺腺癌患者的临床数据。利用肺腺癌患者的生存时间以及状态数据结合HOTAIR及*PTEN*基因的表达情况，通过R软件“survminer”包中的surv_cutpoint函数选择最佳的cut-off值并进行*Kaplan-Meier*生存分析HOTAIR及*PTEN*基因表达与总生存期(overall survival, OS)的相关性。

### 1.11 统计学处理

采用SPSS 17.0统计软件进行数据分析，计量资料以均数±标准差(Mean±SD)表示，采用*t*检验，计数资料比较采用χ^2^检验，*P* < 0.05表示差异有统计学意义。HOTAIR及PTEN表达与肺腺癌预后的关系采用*Kaplan-Meier*模型分析及*Log-rank*检验法，以*P* < 0.05为差异有统计学意义。

## 2 结果

### 2.1 HOTAIR在HCC827GR耐药细胞中表达增高

RT-qPCR结果显示HOTAIR在HCC827GR耐药细胞中表达增高(*P* < 0.01，图 1)，提示HOTAIR可能参与介导吉非替尼耐药。

**1:**
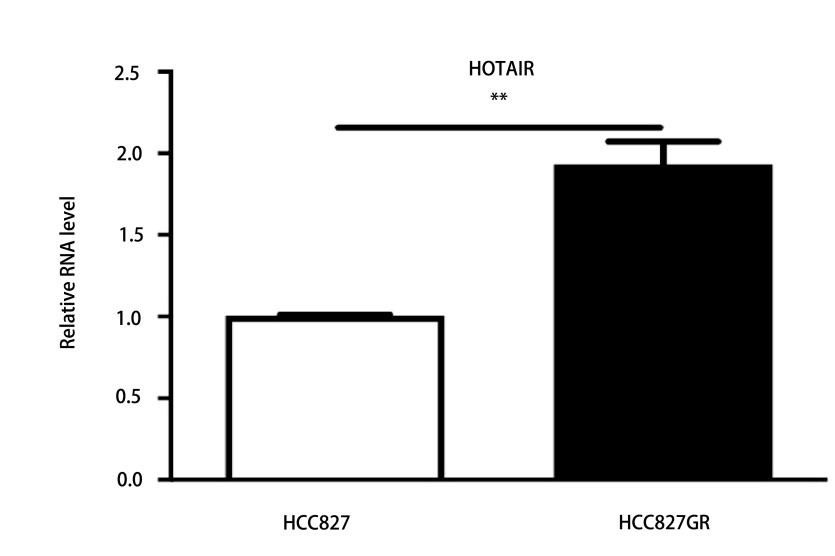
HOTAIR在HCC827GR耐药细胞中表达增高。 The expression of HOTAIR increased in HCC827GR cells. ^**^P < 0.01

### 2.2 下调HOTAIR后HCC827GR细胞中HOTAIR表达降低

RT-qPCR结果显示，HCC827GR/HOTAIR-NC组与HCC827GR组相比无统计学差异，HCC827GR/si-HOTAIR组与HCC827GR/HOTAIR-NC组相比，转染后HCC827GR细胞中HOTAIR mRNA的表达水平下降(70.16±0.57)%，有显著差异(*P* < 0.01，图 2)。结果提示si-HOTAIR转染可显著降低细胞中内源表达的HOTAIR。

**2:**
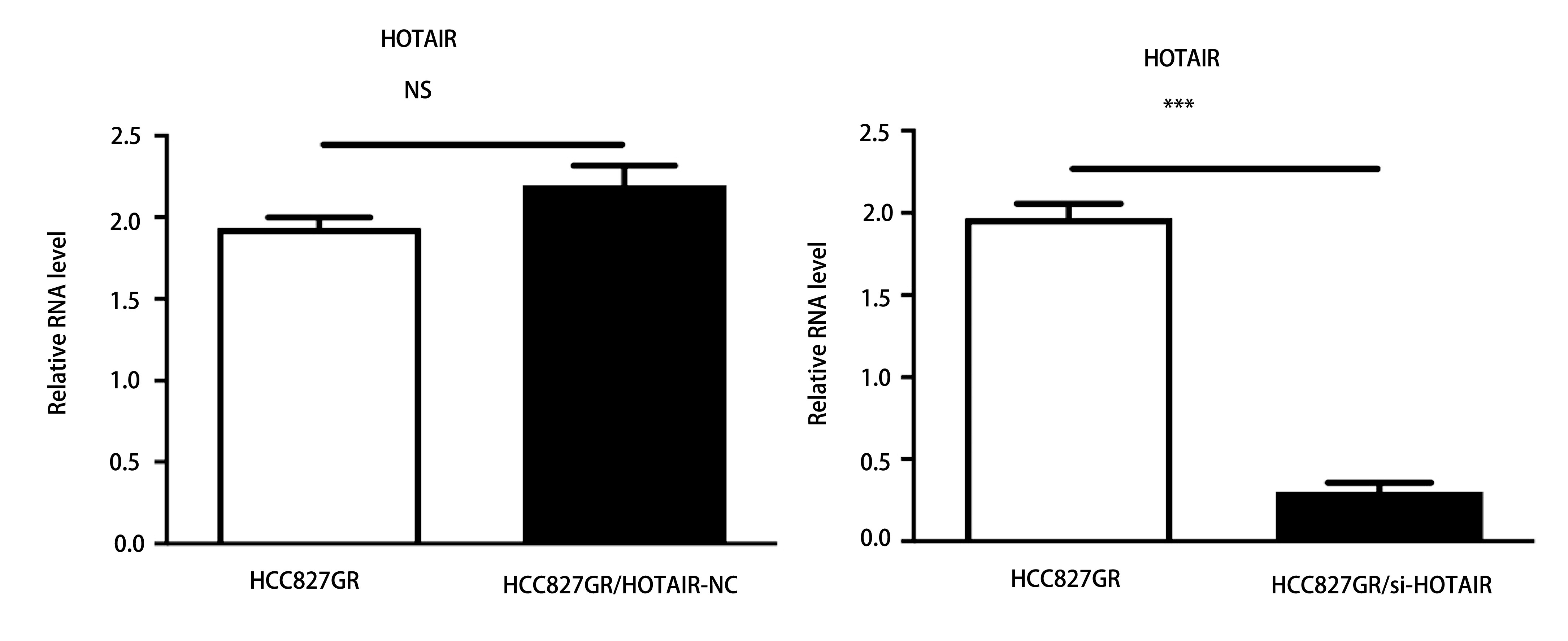
下调HOTAIR后HCC827GR细胞中HOTAIR表达降低。 The expression of HOTAIR decreased in HCC827GR cells after down-regulation of HOTAIR. ^***^P < 0.001; NS: no significant.

### 2.3 下调HOTAIR逆转HCC827GR细胞对吉非替尼的耐药

MTT检测结果显示吉非替尼对转染前后HCC827GR细胞杀伤作用均随时间及浓度递增，转染后HCC827GR细胞48 h的IC_50_为(3.15±0.08)μmol/L，较转染前(18.14±0.17)μmol/L明显降低(*P* < 0.01，图 3)。

**3:**
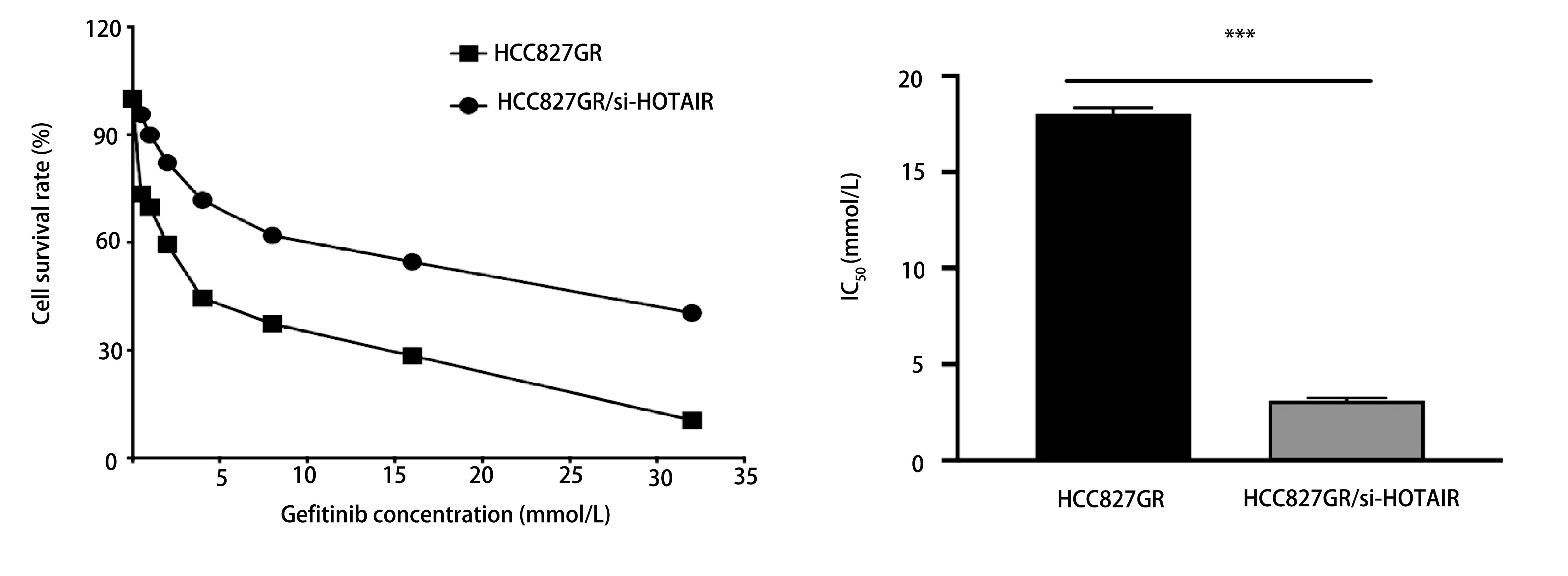
下调HOTAIR逆转HCC827GR细胞对吉非替尼的耐药 Down-regulation of HOTAIR increased the sensitivity of HCC827GR cells to gefitinib. IC_50_: 50% inhibitory concentration.

### 2.4 下调HOTAIR可提高HCC827GR细胞PTEN表达、降低PI3K及AKT表达

RT-qPCR及Western blot法检测分析结果均显示，与HCC827细胞组相比，HCC827GR细胞中PTEN表达水平降低，PI3K及AKT水平升高(*P* < 0.05)。下调HOTAIR后的HCC827GR细胞，相对于阴性对照组，细胞中PTEN的表达水平升高，而PI3K及AKT的表达则较前下降，有显著差异(*P* < 0.01，图 4)。

**4:**
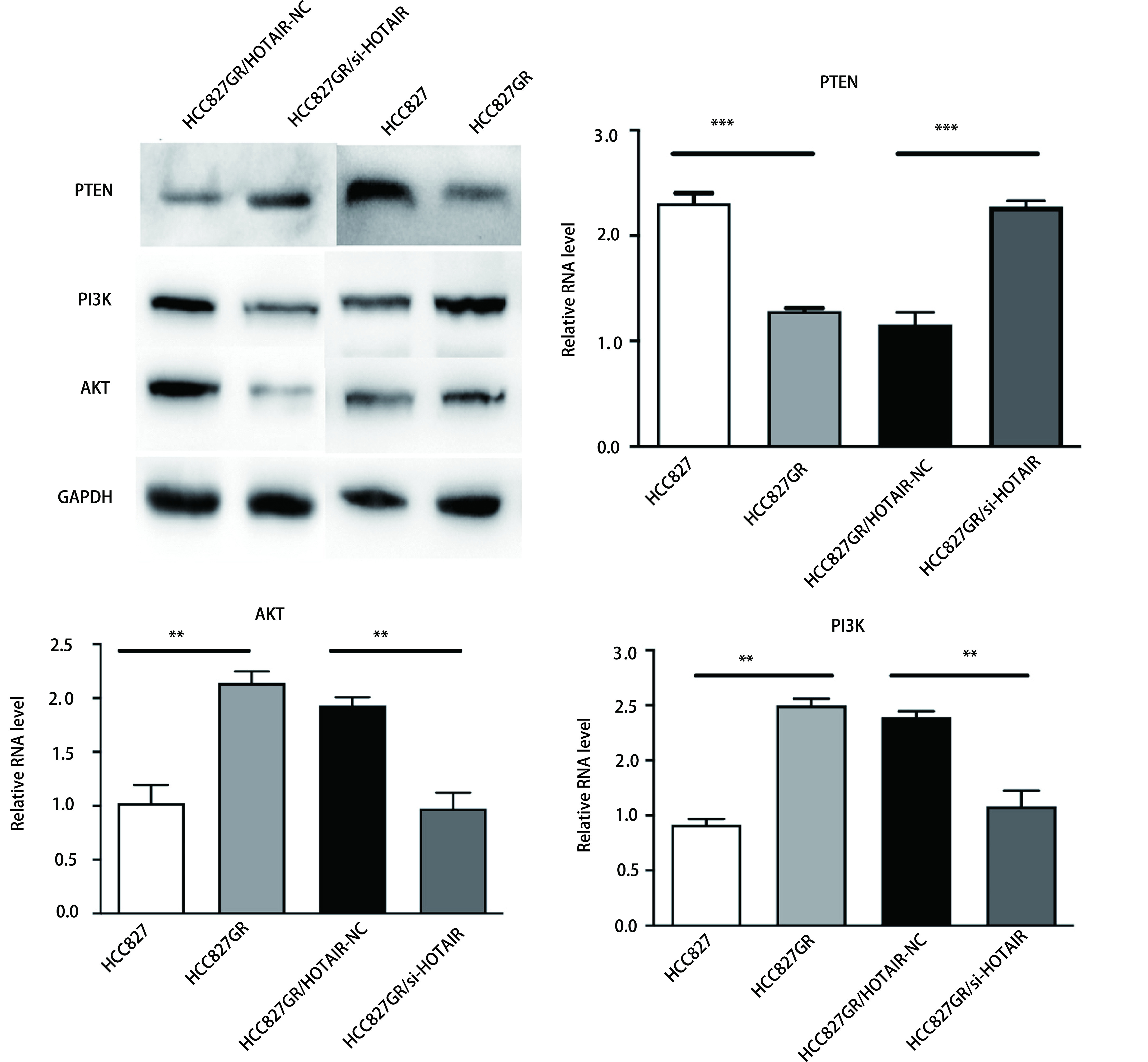
下调HOTAIR可提高HCC827GR细胞PTEN表达，降低PI3K及AKT表达 Down-regulation of HOTAIR can increase the expression of PTEN in HCC827GR cells, and decrease the expression of PI3K and AKT.

### 2.5 下调HOTAIR对HCC827GR细胞凋亡及周期的影响

我们用流式细胞仪技术对HCC827细胞、下调HOTAIR后的HCC827GR细胞以及吉非替尼处理过的HCC827GR细胞进行了细胞凋亡的检测。其中将Annexin V阳性7AAD阴性的细胞为凋亡早期细胞，Annexin V和7AAD双阳性的细胞为凋亡晚期或坏死细胞。分析前上机细胞均进行了细胞计数，凋亡细胞参数设为1万。结果显示，与HCC827细胞相比，HCC827GR细胞凋亡率升高，转染组的HCC827GR细胞凋亡率较空白对照组升高(*P* < 0.05)，吉非替尼联合转染组的HCC827GR细胞凋亡率较单药吉非替尼处理组明显升高(*P* < 0.05，图 5)。HCC827GR细胞中G_0_期/G_1_期细胞的含量较低，S期细胞含量较高，而下调HOTAIR能提高细胞周期中G_0_期/G_1_期细胞的含量，与阴性对照组相比，能够抑制HCC827GR细胞从G_0_期/G_1_期向S期转变，发生G_0_期/G_1_期细胞周期阻滞(*P* < 0.05，图 6)。

**5:**
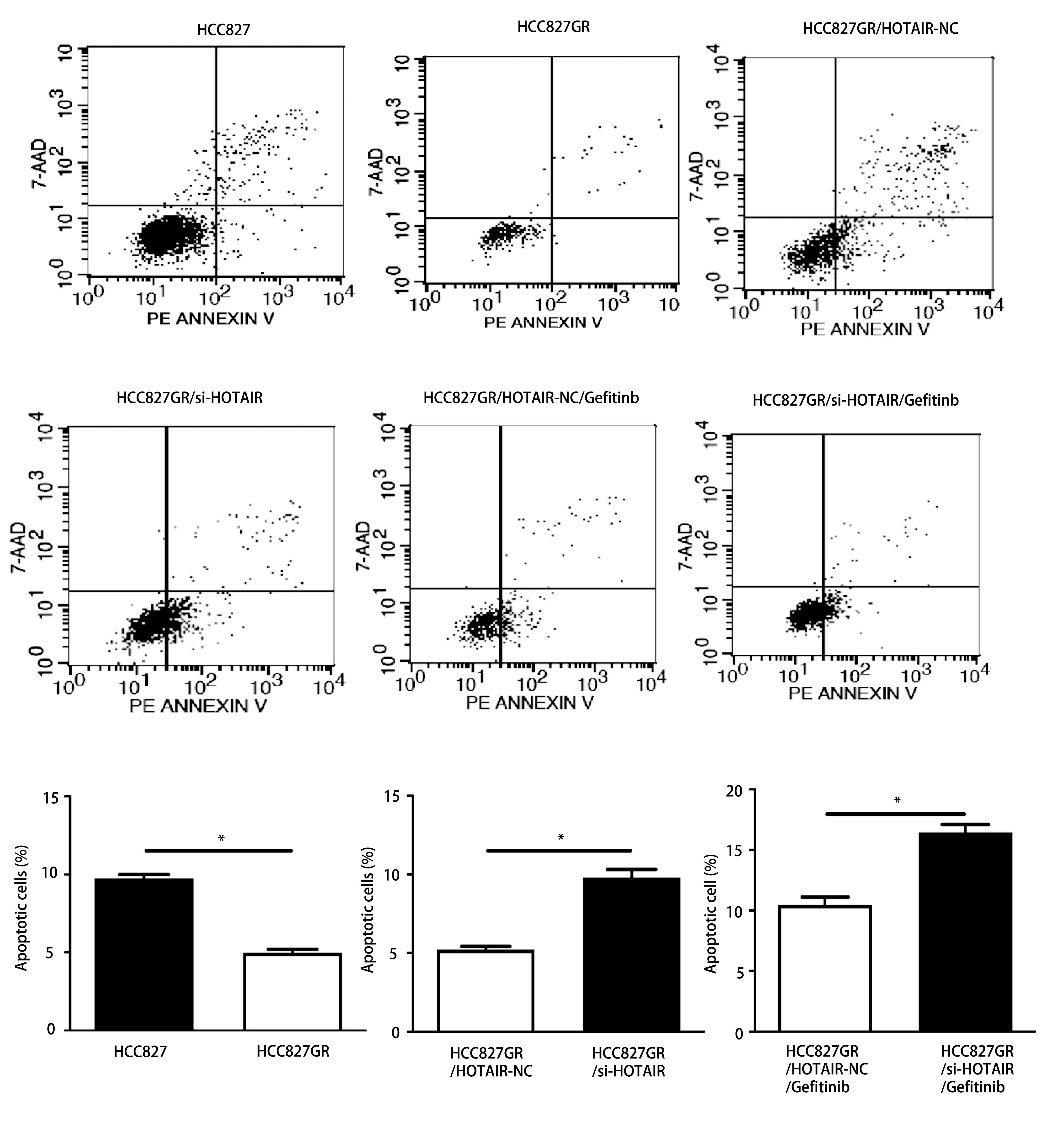
下调HOTAIR促进HCC827GR细胞凋亡 Down-regulation of HOTAIR promotes the apoptosis of HCC827GR. *P < 0.05

**6:**
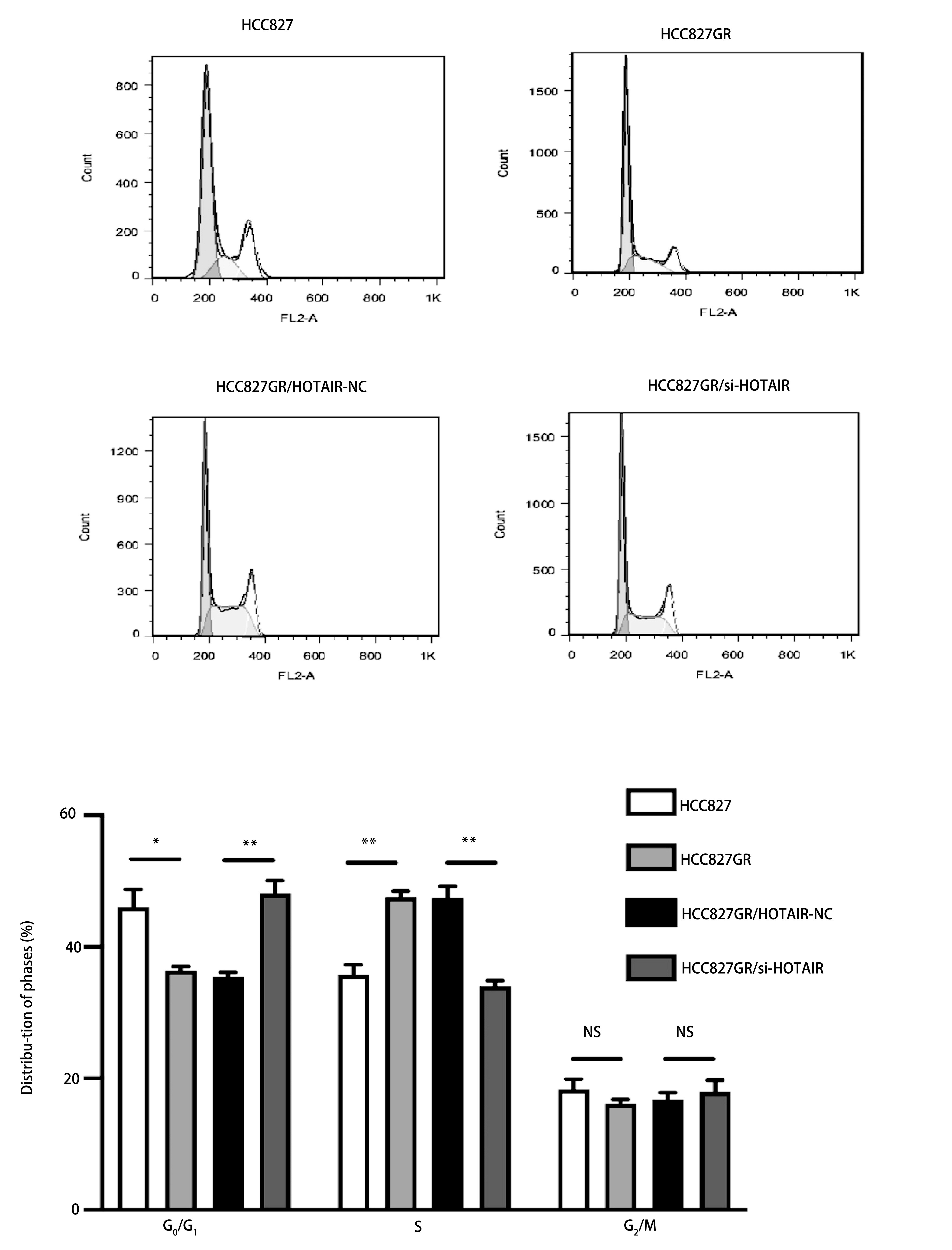
下调HOTAIR抑制HCC827GR细胞增殖(*P* < 0.05) Down-regulation of HOTAIR decreases the cell proliferation ability of HCC827GR (*P* < 0.05)

### 2.6 吉非替尼耐药后NSCLC患者血清HOTAIR表达水平升高

RT-qPCR结果显示吉非替尼耐药后NSCLC患者血清中HOTAIR的表达水平明显高于吉非替尼治疗有效的NSCLC患者(*P* < 0.05，图 7)。

**7:**
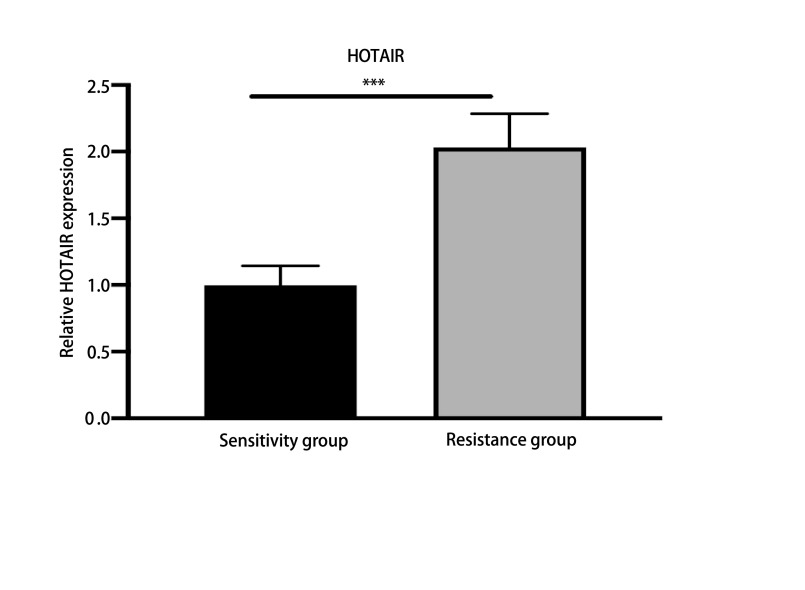
吉非替尼耐药的NSCLC患者血清中HOTAIR表达升高(*P* < 0.05) The expression of HOTAIR increased in the serum of NSCLC patients with gefitinib resis-tance (*P* < 0.05)

### 2.7 PTEN及HOTAIR表达与肺腺癌患者的生存的相关性

*Kaplan-Meier* Plotter生存分析结果表明肺腺癌患者的OS与PTEN表达呈正相关，而与HOTAIR表达则呈负相关(*P* < 0.05，图 8)。

**8:**
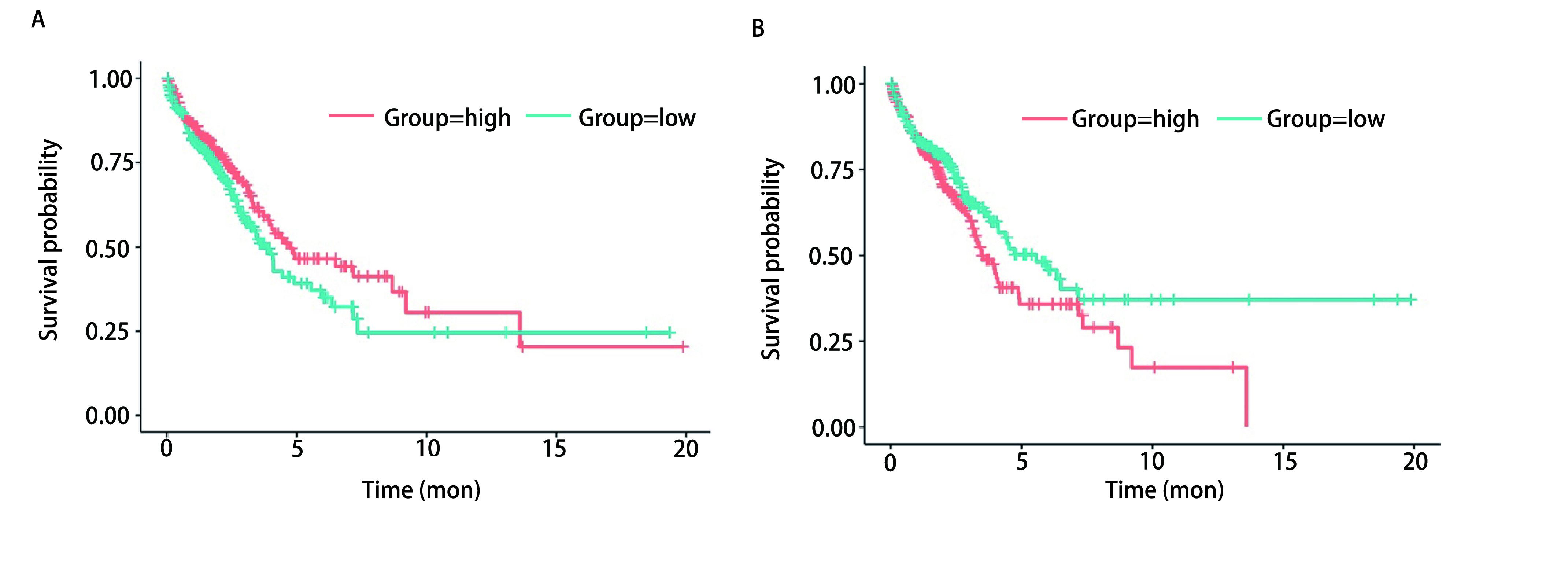
PTEN(A)及HOTAIR(B)表达与肺腺癌患者的生存的相关性 Correlation between the expression of PTEN(A) or HOTAIR(B) and the survival of patients with lung adenocarcinoma

## 3 讨论

吉非替尼是首个口服的EGFR-TKIs，2008年IPASS研究结果^[14]^显示，在腺癌、不吸烟或已戒烟的轻度吸烟者的亚裔晚期NSCLC患者的一线治疗中，与紫杉醇联合卡铂全身化疗相比，吉非替尼组的PFS显著优于化疗组，两组OS无显著差异。NEJGSG002研究^[15]^证实了IPASS的结果，并提出对于*EGFR*突变阳性的NSCLC患者，吉非替尼一线治疗优于标准方案化疗。然而在临床中，存在*EGFR*突变的NSCLC患者吉非替尼的有效维持时间也仅为8个月-10个月，且多数患者容易出现复发，提示此类药物存在较严重的获得性耐药。

虽然大部分的的EGFR-TKIs获得性耐药可用T790M突变及*c-MET*癌基因的扩增解释，但仍有约30%患者的耐药原因尚不明确^[16]^。HCC827是*EGFR*基因19del突变且对吉非替尼敏感的人NSCLC细胞株，我们的前期研究不仅成功构建了吉非替尼耐药细胞株HCC827GR，并且发现*PTEN*基因与肺癌发生发展有着密切的联系，下调PTEN表达可以诱导肺腺癌HCC827细胞对EGFR-TKIs耐药^[17]^，另有研究^[18]^显示在NSCLC中，*PTEN*作为ATK通路上游重要的抑癌基因，可阻止ATK通路的活化，从而阻断ATK调控的下游信号转导，PTEN可能是通过激活PI3K/AKT而减少具有*EGFR*突变的细胞的凋亡，提示PTEN缺失可能是导致非小细胞肺癌患者对EGFR-TKIs发生耐药的原因之一，但其具体分子机制仍尚不明确。

近年来新的研究发现，lncRNA可以发挥癌基因及抑癌基因的作用，能够调控基因转录、转录后和表观遗传水平，与调节染色体的蛋白不同，lncRNA发挥作用不必入核，多以顺式调节方式发挥作用。由于lncRNA可以来源于增强子，故其也有增强子样的作用，同时lncRNA在胞浆参与调节mRNA，并可通过表观遗传学调控改变细胞结构和功能状态^[19]^。大量研究^[20-22]^表明多种lncRNA参与了肺癌的发生发展，并与肺癌患者的预后及对化疗药物的耐药有密切的关系。HOTAIR是第一个被发现的反义转录lncRNA，可通过招募PRC2使相应组蛋白发生甲基化，进而调控有关侵袭转移基因的表达进而参与癌症的进展^[6-7, 23]^。Nakagawa等^[24]^发现NSCLC患者肿瘤组织中HOTAIR的表达水平与肿瘤的大小、分化程度及分期呈正相关。我们的研究发现HOTAIR在HCC827GR耐药细胞及吉非替尼耐药患者血清中表达均增高，同时我们还发现下调HOTAIR后，HCC827GR细胞的增殖能力下降，凋亡率升高，并可引起G_0_期/G_1_期细胞周期阻滞，同时能够使HCC827GR细胞恢复对吉非替尼的敏感性，提示HOTAIR可能参与介导了NSCLC的吉非替尼耐药。既往已有多项研究提示HOTAIR在子宫内膜癌、喉部鳞癌及乳腺癌中可通过靶向调控PTEN/PI3K/AKT通路促进肿瘤的增殖和转移。Ma等^[25]^发现HOTAIR能够和miR-130a结合，通过下调miR-130a导致PTEN等基因下调，进而激活AKT信号通路影响肿瘤的恶性表型。我们通过生物信息学分析预测到HOTARI可作为一种竞争性内源性RNA(competing endogenous RNA, ceRNA)与miR-526b-3p、miR-519d-3p及miR106b-5p相互作用，形成ceRNA的竞争机制，而*PTEN*为miR-526b-3p、miR-519d-3p及miR106b-5p的下游靶基因，提示高表达HOTAIR可导致miRNA对PTEN的抑制力减弱，这与既往多项研究结果不符，考虑因数据库纳入样本量不足及疾病种类不同而此造成偏差。在本研究中，我们也发现下调HOTAIR后，HCC827GR细胞中PTEN表达升高，而PI3K及AKT的表达则较前下降，同时我们从TCGA数据库中下载肺腺癌患者的临床数据，通过生存分析发现肺腺癌患者总生存与PTEN表达呈正相关，而与HOTAIR表达则呈负相关，这与我们的实验研究结果相一致。因此我们推测，HOTAIR可调节PTEN/PI3K/AKT信号通路，通过负反馈作用于PTEN，调控肿瘤细胞的增殖及凋亡，下调HOTAIR表达可以逆转HCC827GR细胞对吉非替尼的耐药。

本研究首次证明了HOTAIR与HCC827细胞对吉非替尼耐药之间的关系及其机制，提示HOTAIR的表达可有助于判断NSCLC患者对EGFR-TKIs类药物的治疗反应，并为NSCLC靶向治疗耐药后的选择提供了新的方向。

**Author contributions**

Zhai Y, Li LN and Chen Q conceived and designed the study. Zhai Y and Chen Q performed the experiments. Wang YZ and Li X analyzed the data. Zhai Y and Li LN contributed analysis tools. Zhai Y, Li LN and Chen Q provided critical inputs on design, analysis, and interpretation of the study. All the authors had access to the data. All authors read and approved the final manuscript as submitted.
